# Effect of Blast-Furnace Slag Replacement Ratio and Curing Method on Pore Structure Change after Carbonation on Cement Paste

**DOI:** 10.3390/ma13214787

**Published:** 2020-10-27

**Authors:** Junho Kim, Seunghyun Na, Yukio Hama

**Affiliations:** 1 Department of Architecture, National Institute of Technology, Oyama College, Nakakuki, Oyama-shi, Tochigi 323-0806, Japan; kim@oyama-ct.ac.jp; 2 Institute of Industrial Science, The University of Tokyo, 4-6-1 Komaba, Meguro-ku, Tokyo 153-8505, Japan; nash1122@naver.com; 3 College of Environmental Technology, Graduate School of Engineering, Muroran Institute of Technology, 27-1 Mizumoto, Muroran 050-8585, Japan

**Keywords:** blast-furnace slag, carbonation, pore structure

## Abstract

The frost damage resistance of blast-furnace slag (BFS) cement is affected by carbonation. Hence, this study investigates the carbonation properties of pastes incorporating BFS with different replacement ratios, such as 15%, 45%, and 65% by weight, and different curing conditions, including air and carbonation. The BFS replacement ratio properties, determined by the Ca/Si ratio of calcium silicate hydrate in the cement paste sample, were experimentally investigated using mercury intrusion porosimetry, X-ray diffraction, and thermal analysis. The experimental investigation of the pore structure revealed that total porosity decreased after carbonation. In addition, the porosity decreased at a higher rate as the BFS replacement rate increased. Results obtained from this study show that the chemical change led to the higher replacement rate of BFS, which produced a higher amount of vaterite. In addition, the lower the Ca/Si ratio, the higher the amount of calcium carbonate originating from calcium silicate hydrate rather than from calcium hydroxide. As a result of the pore structure change, the number of ink-bottle pores was remarkably reduced by carbonation. Comparing the pore structure change in air-cured and carbonation test specimens, it was found that as the replacement rate of BFS increased, the number of pores with a diameter of 100 nm or more also increased. The higher the replacement rate of BFS, the higher the amount of calcium carbonate produced compared with the amount of calcium hydroxide produced during water curing. Due to the generation of calcium carbonate and the change in pores, the overall number of pores decreased as the amount of calcium carbonate increased.

## 1. Introduction

The Ca/Si ratio of C–S–H (calcium silicate hydrate) affects the properties of cement and cementitious materials. When the amount of blast-furnace slag (BFS) increases, the Ca/Si ratio of C–S–H in the cement composite decreases. Many studies have focused on the characteristics of the Ca/Si ratio of C–S–H. Tanaka et al. [1] found that the density of C–S–H is proportional to the Ca/Si ratio, and it is not related to the environment of C–S–H hydration. Ishida et al. [2] reported that the Ca/Si ratio did not affect the carbonation speed.

The Ca/Si ratio has an impact even after curing. In particular, concrete shows a low resistance to carbonation in an accelerated environment caused by a lower Ca/Si ratio. Many researchers have studied the relationship between compressive strength and durability during the carbonation of cement. In previous studies [3,4,5], the carbonation of concrete was examined, and it was found that the pore structure dramatically changed after carbonation. In addition, Phung et al. [6] found that the carbonation-induced change affects the microstructure of the concrete and decreases its water permeability. Borges et al. [7] revealed that the durability of concrete increases as the curing temperature increases and that the porosity within the cement mixture decreases after carbonation, whereas the density of the cement paste increases. Rostami et al. [8] reported that carbonation improves strength and durability. Zhang et al. [9] experimentally identified the resistance to freeze–thaw with and without carbonation, and the results revealed that the reduction in weight loss is due to carbonation. However, the effects of the change in chemical composition have not been considered. Therefore, it is necessary to determine the effects of the change in chemical composition on pore structure.

In this study, which aims to determine the suitable pore structure involved in the carbonation of BFS-blended cementitious composites, the effects of different pore structures and hydration products on the carbonation of cement paste samples are studied using Mercury Intrusion Porosimetry (MIP), X-ray diffraction (XRD) analysis, and thermal analysis (TG-DTA). The results obtained from this study provide invaluable information regarding the durability of cementitious composites, offering a better understanding of the carbonation of BFS-blended cementitious composites.

## 2. Experimental Program

### 2.1. Experimental Materials and Sample Preparation

The cement used in this experiment was Ordinary Portland Cement (OPC). OPC is denoted as N with a density of 3.17 g/cm^3^. In addition, BFS with a fineness of 3930 cm^2^/g (density of 2.91 g/cm^3^) was used to replace OPC at 15%, 45%, and 65% by weight. All paste specimens were cast into 50 × 100 mm plastic cylinder molds with a water–binder ratio (W/B) of 0.65 without any chemical admixture. The chemical composition and physical properties of material are shown in Table 1. The mixture proportions of the paste and the dosage of mineral admixtures are shown in Table 2. Pore size distribution was determined using the mercury intrusion method and chemical composition by the XRD method. DTG (differential thermogravimetry) was used to conduct a thermal analysis. The total number of pores was also measured.

### 2.2. Experimental Method

#### 2.2.1. Mercury Intrusion Porosimetry

The pore size distribution of the samples was determined by the MIP method, which is a method commonly used to measure the pore size distribution in cementitious composite materials [10,11,12]. The surface tension of the mercury and the mercury density were 0.480 N/m and 13.546 g/mL, respectively, assuming a 140° contact angle. The samples were exposed to CO_2_ in the control chamber before measurements were taken from the carbonated cement paste. The MIP technique requires that the hardened cementitious composite materials are thoroughly treated to remove water and evacuated prior to testing. After the curing, the cement paste samples were cut into 5 mm^3^ cubes. Acetone was used to halt the cement paste hydration reaction, and a vacuum drying treatment was administered to each sample. The pore size of each sample was measured using a porosimeter (Autopore Master33, Quantachrome instruments, Tokyo, Japan), in which a hydraulic pump was used to generate the pressure and a contact sensor was used to measure the volume of mercury. It has been sufficiently demonstrated that the effect of a sample’s mass and size on the pore structure result is relatively small [13]. Moreover, the MIP measurement is highly dependent on the equipment. According to the manufacturer of the porosimeter equipment used in this study, the accuracy of this equipment in the range of 0–220 MPa is ±0.11% and the linearity in the range from 0 to maximum pressure is ±0.05%, which also reveals that the measurement results in this study are reliable.

#### 2.2.2. X-ray Diffraction

An analysis of the hydration was conducted using the XRD method to assess various cement hydration products. The XRD characteristics and ignition loss (LOI) characteristics were measured for each curing age condition, acetone replacement was carried out to stop the hydration reaction, and the samples were dried before the measurements were taken.

Prior to XRD analysis, the samples were dried using vacuum drying equipment for 24 h. The samples were then turned into powder using a vibration mill, and the powder samples were used to conduct the XRD analysis. For the analysis, a semiconductor-type high-speed detector was used with CuKα as a target, a tube current of 40 mA, a tube voltage of 45 kV, a step width of 0.02 degrees, and a 2θ scan range of 5–70 °C.

#### 2.2.3. Thermal Analysis

The powder samples were used a cement paste. The heating rate was 30–1000 °C (20 °C/min) under a nitrogen atmosphere. The reduction in weight was measured based on the amounts of calcium carbonate (CC) and calcium hydroxide (CH). The CO_2_ loss in the carbonation range of 600–750 °C was converted into a CH loss, and the CH was calculated in the dihydroxylation range of 400–500 °C. Borges suggested how to measure the amounts of CH and CC, Equations (1)–(6). [7]

(1) $$\begin{array}{c} \mathrm{Ca}{\left(\mathrm{OH}\right)}_{2}\\ 74\;g/\mathrm{mol} \end{array}+\begin{array}{c} {\mathrm{CO}}_{2}\\ 44\;g/\mathrm{mol} \end{array}\to \begin{array}{c} {\mathrm{CaCO}}_{3}\\ 100\;g/\mathrm{mol} \end{array}+\begin{array}{c} H_{2}O\\ 18\;g/\mathrm{mol} \end{array}$$

(2) $$\%\mathrm{CC}=\frac{{\mathrm{dc}}_{\mathrm{loss}}\cdot 100}{44}=2.27\cdot {\mathrm{dc}}_{\mathrm{loss}}$$

(3) $${\%\mathrm{CH}}_{\mathrm{dc}-\mathrm{loss}}=\frac{\%\mathrm{CC}\cdot 74}{100}=\frac{2.27\cdot {\mathrm{dc}}_{\mathrm{loss}}\cdot 74}{100}=1.68\cdot {\mathrm{dc}}_{\mathrm{loss}}$$

(4) $$\begin{array}{c} \mathrm{Ca}{\left(\mathrm{OH}\right)}_{2}\\ 74\;g/\mathrm{mol} \end{array}\to \begin{array}{c} \mathrm{CaO}\\ 56\;g/\mathrm{mol} \end{array}+\begin{array}{c} H_{2}O\\ 18\;g/\mathrm{mol} \end{array}$$

(5) $${\%\mathrm{CH}}_{\mathrm{dh}-\mathrm{loss}}=\frac{{\mathrm{dh}}_{\mathrm{loss}}\cdot 74}{18}=4.11\cdot {\mathrm{dh}}_{\mathrm{loss}}$$

(6) $${\%\mathrm{CH}}_{\mathrm{total}}={\%\mathrm{CH}}_{\mathrm{dc}-\mathrm{loss}}+{\%\mathrm{CH}}_{\mathrm{dh}-\mathrm{loss}}=1.68\cdot {\mathrm{dc}}_{\mathrm{loss}}+4.11\cdot {\mathrm{dh}}_{\mathrm{loss}}$$

The amount of carbonation from CH and C–S–H was calculated based on the amounts of water curing, air curing, and carbonation by using Equations (7) and (8).

(7) $${\%\mathrm{CH}}_{\mathrm{initial}}=\%\mathrm{CH}+\frac{\%\mathrm{CC}\cdot 74\;g/\mathrm{mol}}{100\;g/\mathrm{mol}}=\%\mathrm{CH}+0.74\%\mathrm{CC}=\left(A\right)+0.74\cdot \left(B\right)$$

(8) $$\%\mathrm{CC}=\frac{\%\mathrm{CH}\cdot 100}{74}=1.35\cdot \%\mathrm{CH}=1.35\cdot \left(F\right)$$

### 2.3. Curing Condition

**Figure 1:**
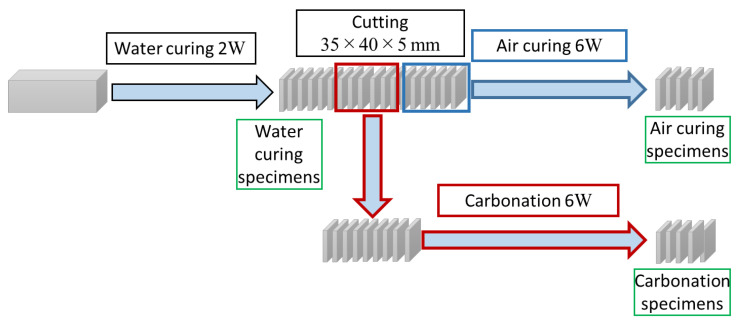
Curing process and sampling process.

Figure 1 shows the curing process and the sampling point in this study. The mortar sample was cut 5 mm from the top in order to avoid the effect of bleeding. The thickness of the test specimen was 5 mm in order to complete the carbonation of the entire set of test samples in a short period of time. The water-cured sample was cured in water for 2 weeks, and the air-cured sample was air cured for 6 weeks after 2 weeks of water curing. In addition, the carbonation sample was subjected to accelerated carbonation (20 °C, RH 60%, and 5% CO_2_) for 6 weeks after 2 weeks of water curing. In addition, the complete carbonation of the test specimen was performed by spraying a phenolphthalein solution, and complete carbonation was confirmed.

## 3. Experiment Result

### 3.1. Characteristics of Chemical Properties According to Carbonation

#### X-ray Diffraction Result

**Figure 2:**
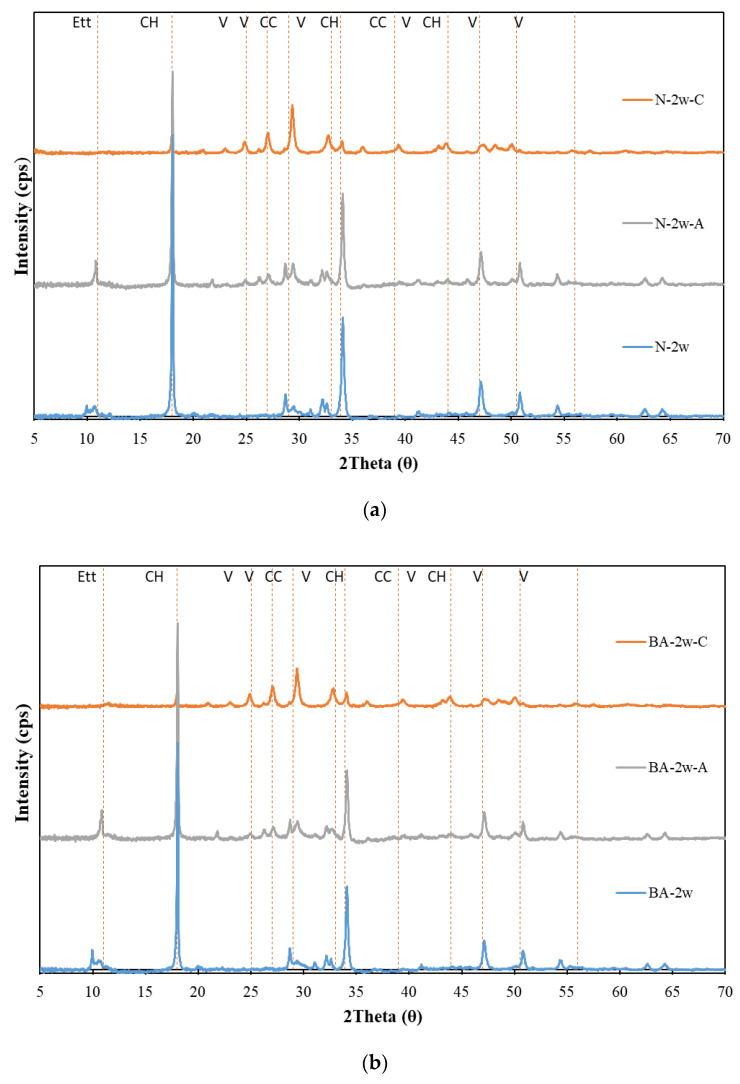

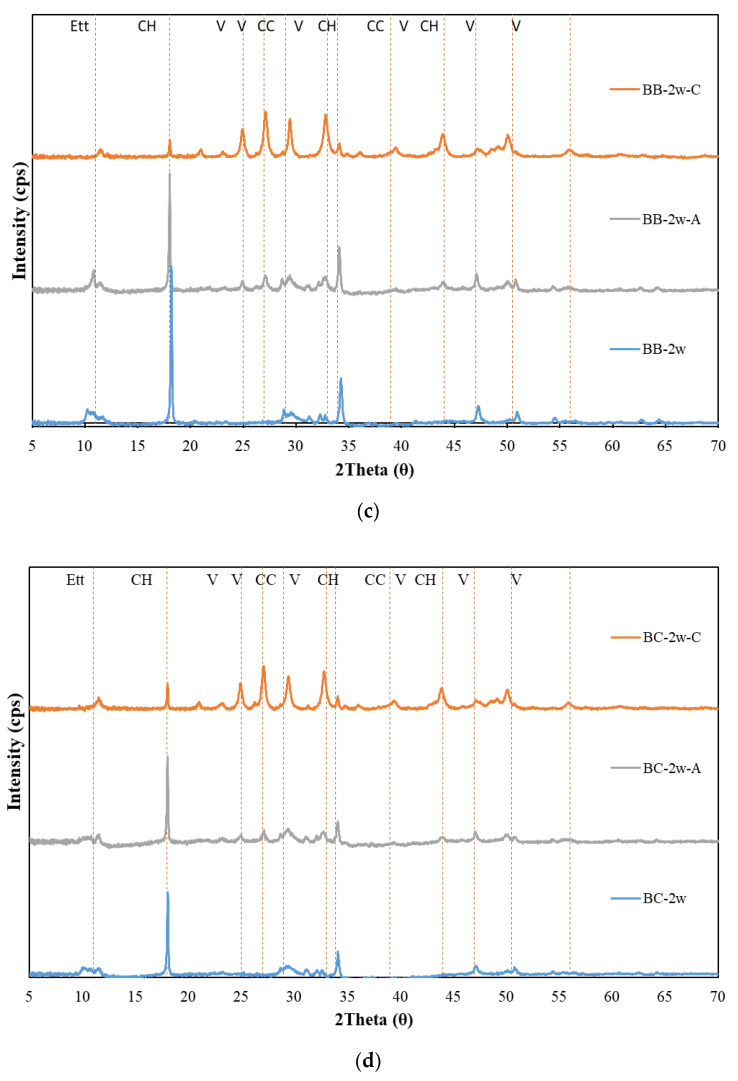
XRD patterns of carbonated cement paste. (**a**) N; (**b**) BA; (**c**) BB and (**d**) BC.

Figure 2 shows the effect of the hydration products on hardened cement paste with BFS replacement ratios of 15%, 45%, and 65% for various cement samples. The XRD data for the paste samples were collected after water curing, air curing, and carbonation, as shown in Figure 2a–d. The hydration products of cement, calcium hydroxide, calcium carbonate, and vaterite were investigated; the peaks of calcium hydroxide (Ca(OH)_2_; CH, peaks of 2θ = 18, 33.9, 47), vaterite (V, peaks of 2θ = 25, 27, 33, 44, 50.5, 56), and calcite (C, peaks of 2θ = 29, 39) were determined. As shown in Figure 2, the CH peak is found in all of the cement paste samples and decreases as the proportion of BFS increases due to the additional hydration of BFS, which reacts with CH and generates the C–S–H gel. This reduction in calcium hydroxide inside of the cement mixed with slag was also observed when air curing was performed. The XRD data of the cement paste sample with BFS after carbonation curing are shown in Figure 2. The peak of the vaterite can more easily be observed with a higher BFS replacement ratio because the generation of vaterite under carbonation results in the decomposition of the C–S–H gel, in agreement with previous studies [7,14].

### 3.2. Characteristics of the Amount of Calcium Hydroxide and Calcium Carbonate after Carbonation

#### 3.2.1. DTG Curves of Calcium Hydroxide and Calcium Carbonate

**Figure 3:**
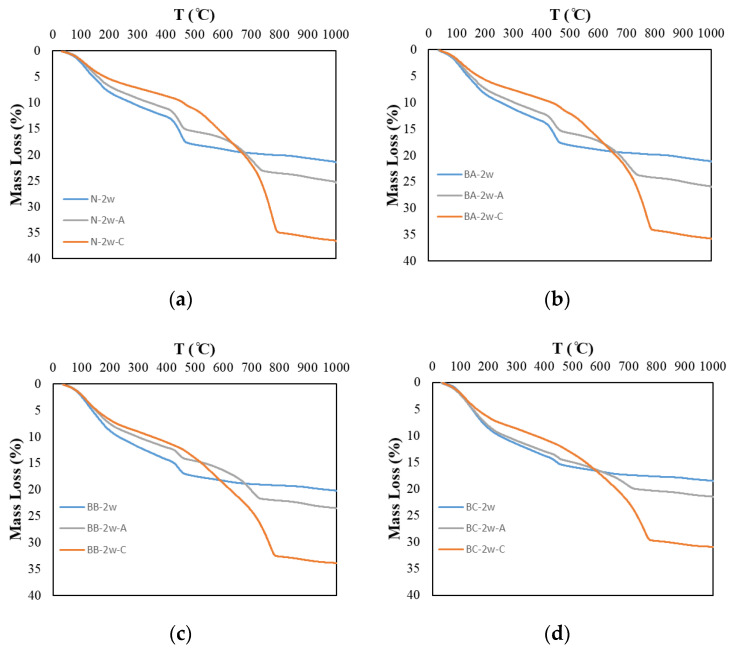
Mass loss of carbonated cement paste. (**a**) N; (**b**) BA; (**c**) BB and (**d**) BC.

**Figure 4:**
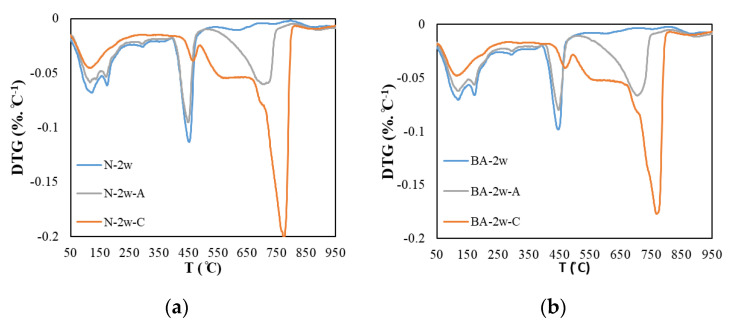

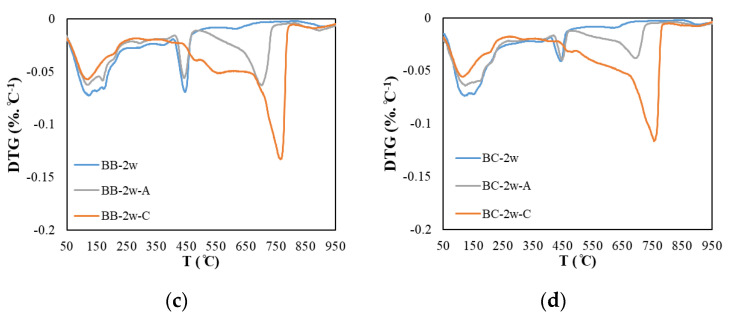
DTG of carbonated cement paste. (**a**) N; (**b**) BA; (**c**) BB and (**d**) BC.

Figure 3 and Figure 4 show the DTG results and mass loss results of the thermal analysis at a curing age of 2 weeks. We focused the investigation on calcium carbonate and calcium hydroxide. The calcium hydroxide exhibits decreased peaks in the range of 400–500 °C and the calcium carbonate exhibits decreased peaks in the range of 600–800 °C. In Figure 4, the mass loss due to calcium hydroxide and calcium carbonate is confirmed. In each temperature range, a weight change owing to calcium hydroxide and calcium carbonate was observed. In addition, in the carbonation specimen, as the replacement rate of BFS increased, the overall mass loss tended to decrease.

In Figure 4, which compares the water-cured specimens, it can be observed that as the replacement rate of BFS increases, the rate of CH formation decreases, as described by Wu. et al. in 2017 [15]. Air-cured specimens also showed the same trend; in this case, carbonation increases the CC peak and decreases the CH peak. It appears that the thickness of the air-cured specimen (5 mm), which was cut after water curing, caused the rate of carbonation to increase.

In comparing the carbonation test samples, almost no CH was found in the BB and BC test samples, and the CC peak of the carbonation test sample was larger than that of the water-cured and air-cured test samples. In addition, the results show that as the BFS replacement rate increases, the CC generation rate decreases. These results show the same trend as previous studies, and because the amount of CC varies according to the amount of CH, it is determined that the amount of CC produced by the test specimen, given the small amount of CH produced during water curing, is also small [15].

#### 3.2.2. The Amount of Calcium Hydroxide and Calcium Carbonate

**Figure 5:**
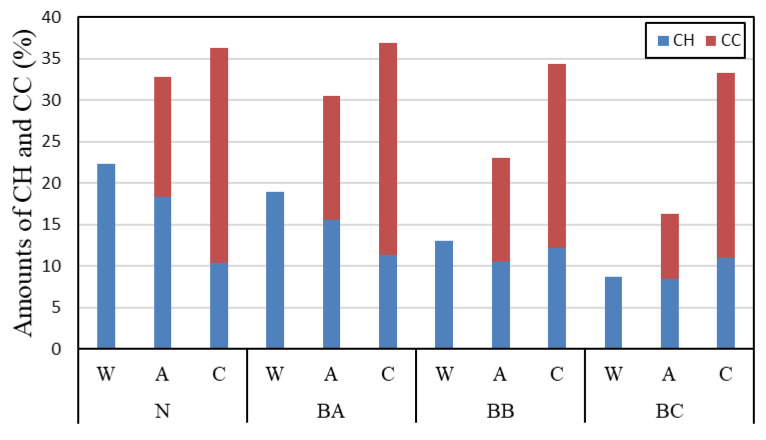
Amount of calcium hydrate (CH) and calcium carbonate (CC) in carbonated cement paste.

**Figure 6:**
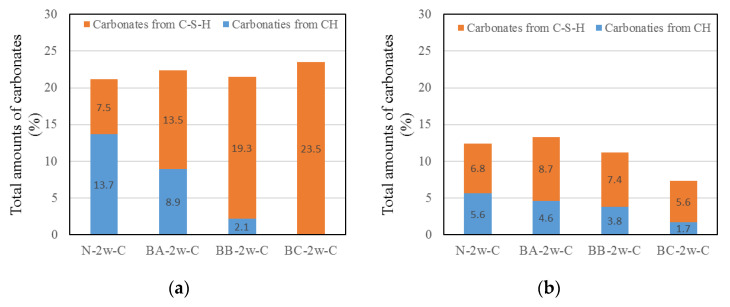
Carbonation of CH and calcium silicate hydrate (C–S–H) of the paste samples with different replacement ratios of blast-furnace slag (BFS). (**a**) Comparison of water curing and carbonation samples and (**b**) Comparison of air curing and carbonation samples.

Figure 5 shows the amount of calcium hydroxide (CH) and calcium carbonate (CC) in BFS-blended paste samples determined by thermal analysis at a curing age of 2 weeks. The replacement ratios of the BFS-blended cement paste samples included ratios of 15%, 45%, and 65% by weight of cement. As shown in Figure 6, the amount of CH in the paste samples tends to decrease as the BFS replacement ratio increases because of the decreasing cement content. In the case of air curing, the amount of CH and CC tends to decrease according to the BFS replacement rate. In the case of carbonation, regardless of the replacement rate of BFS, a similar amount of CH was produced. In the case of CC, BB, and BC, which had a high BFS replacement rate, the amount of CH produced was slightly lower. However, when considering the amount of CH generated during underwater curing and air curing, the amount of CC produced is higher than expected. This result is in agreement with previous studies, and in the specimens with a high replacement rate of BFS, it is determined that the carbonation amount by C–S–H is greater than that by CH. [15] The same tendency was found in a previous study where a test specimen was substituted with fly ash. It is considered that this tendency occurs when C–S–H with a low Ca/Si ratio is produced [16].

#### 3.2.3. Carbonation of CH and C–S–H

Figure 6 shows the carbonation of CH and C–S–H in BFS-blended paste samples at a water curing age of 2 weeks. The extent of carbonation of CH and C–S–H during air curing and after carbonation in the paste samples is also shown. Figure 6a shows the calculated results of the water-cured and carbonated paste samples. The results show that the carbonates from C–S–H increase as the replacement ratio of BFS increases. In the case of the BC-2w-C paste samples, most of the carbonates formed in these samples were from C–S–H. The obtained results align with those of previous studies reported in the literature [8,16,17]. Figure 6b shows the calculated result of the amount of carbonate from CH and C–S–H in the water-cured and air-cured paste samples. The total carbonation amount of the paste samples tends to decrease as the BFS replacement ratio increases. In the carbonation amount from C–S–H, there is a slight difference. However, when the replacement rate of BFS increases, the carbonation amount from CH decreases [15,16].

### 3.3. Characteristics of Pore Structure after Carbonation

#### 3.3.1. Pore Volume of Log Differential Results

**Figure 7:**
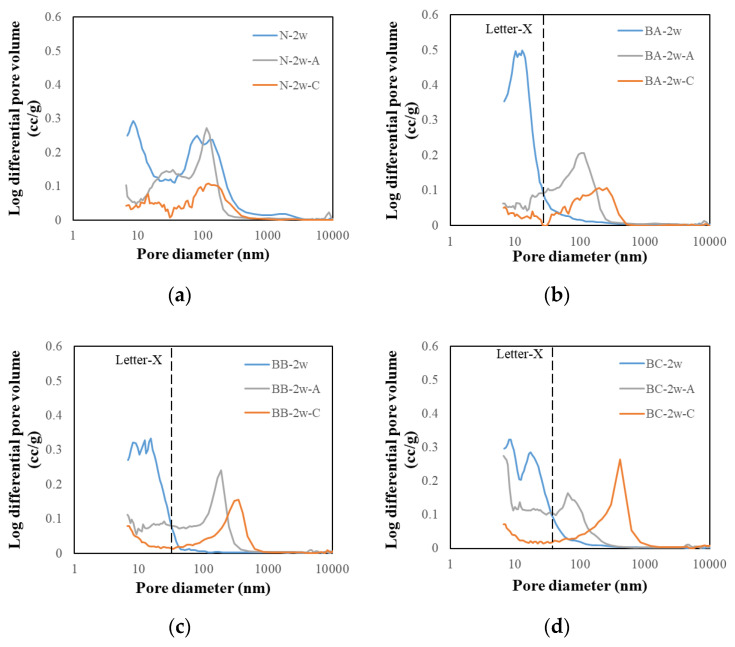
Pore size distributions of carbonated cement paste. (**a**) N; (**b**) BA; (**c**) BB and (**d**) BC.

Figure 7 shows the change in pore size distribution of the BFS-blended paste samples at a water curing age of 2 weeks. In previous studies, the effect of void structure on frost resistance was studied [17,18]. Therefore, it is expected that the void structure changed by carbonation will affect the durability. Figure 7a shows the pore change results for the N samples’ carbonation (6 weeks), air curing (6 weeks), and water curing (2 weeks), respectively. It can be observed that the pore volume of the small pores (diameters of approximately 80 nm) decreased as the air curing progressed, indicating that the pore structure changed according to curing age. In addition, the carbonation paste samples tended to decrease in comparison with the air-cured samples. In both the water-cured and the air-cured samples, the size of the small pores decreased and the size of the pores with diameters greater than 100 nm increased. This change in the pore structure is considered to be an effect of drying the specimen during air curing and after water curing.

Figure 7b–d shows the pore change results for the BFS-blended paste samples. The peak of the pore volume is higher than that of the N paste samples at a low pore diameter, and the pore volume of the small pores decreases after the carbonation progresses, indicating that the pore structure is changed.

These results show the same trend as previous studies, which confirmed that the peaks of the pore volume moved in the direction of the larger pore size due to the carbonation. However, the reason for this is inconclusive and requires further research [19]. In other studies, a shift in pore size of this tendency was observed, but the reason was considered to be the low amount of CH generation [20].

The reason for the shift of the pore peak of the air-cured specimen towards a larger pore size compared with the peak of the water-cured specimen remains unknown. Although verification is necessary, it is possible that the contraction caused by BFS may be the reason.

#### 3.3.2. Cumulative Pore Volume Results

**Figure 8:**
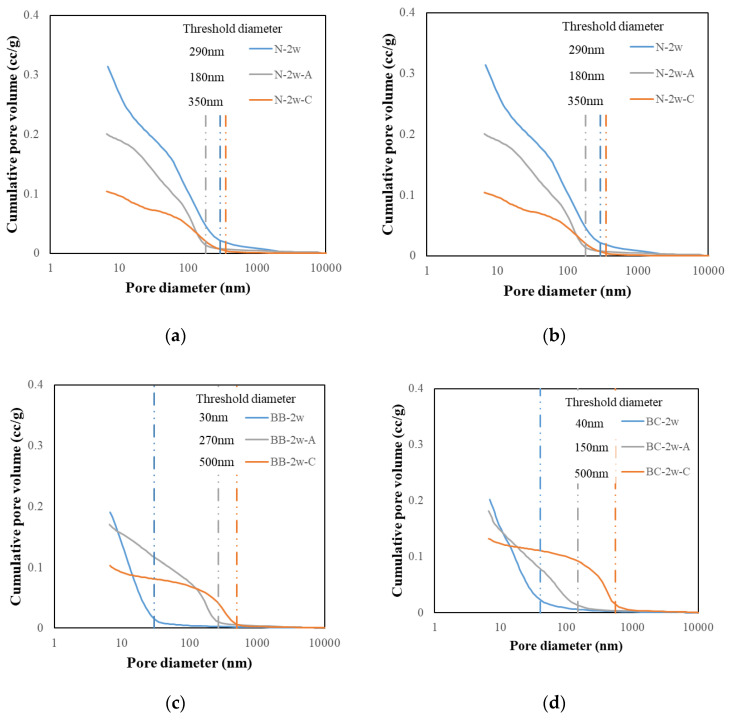
Cumulative pore volume and threshold diameter of carbonated cement paste. (**a**) N; (**b**) BA; (**c**) BB and (**d**) BC.

Figure 8 shows the cumulative pore volume results for the carbonation (6 weeks), air-cured (6 weeks), and water-cured samples (2 weeks). It is well known that the pore volume of cement-based composites depends on the cement hydration. This finding is in good agreement with the BFS-blended paste samples. In fact, cement hydration causes a decrease in the pore volume after carbonation, implying that there is a negligible effect from water curing.

In comparing the air-cured specimen and the carbonated specimen, it was observed that as the replacement rate of BFS increases, the number of pores with a size of 100 nm or larger tends to increase. The letter X is a carbonated specimen that overlaps with the air-cured specimen. The position of the letter X moves to a smaller pore size as the replacement rate of BFS increases; as a result, the number of pores with a size of 100 nm or more tends to increase. Such a trend was also found in previous studies, but in the previous studies, the experiment was not based on the replacement ratio of the binder, as the experiment was performed using fly ash. Hence, there was no consideration of this tendency [20]. In this study, the effect of the replacement ratio of the binder was considered, and the effect of the replacement rate of BFS could be confirmed. Furthermore, based on the XRD results, it is determined that the generation of calcium carbonate of different types indicates this tendency. In particular, vaterite is considered to exhibit such a tendency.

#### 3.3.3. Total Porosity Results

**Figure 9:**
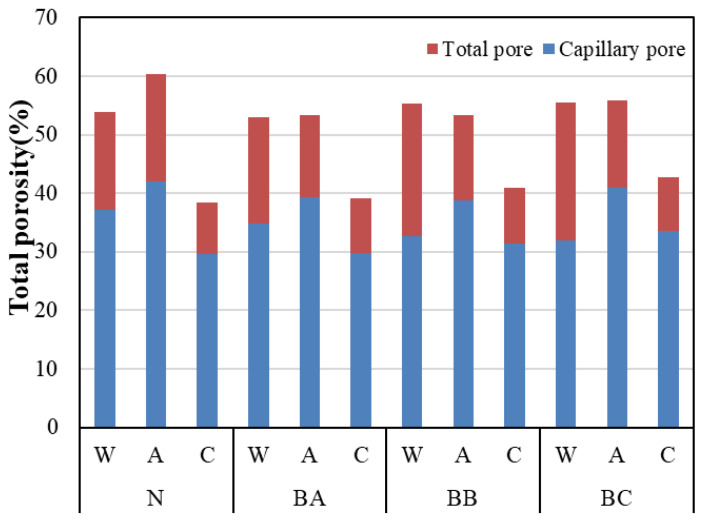
Total pore volume of carbonated cement paste.

Figure 9 shows the total porosity of the BFS-blended paste samples at a water curing age of 2 weeks. The specific volume of the paste samples is shown in Table 3. The method of measuring the total porosity of the samples was carried out in the same way as in the previous paper [21]. A vacuum was created to fill the test body full of water. The weight dried at 40 °C for 2 days were the capillary pores, and the weight dried at 105 °C for 1 day were the gel pores. In addition, the weight change after drying at 105 °C was measured as the total porosity [21]. Figure 9 is the total porosity for the carbonated (6 weeks), air-cured (6 weeks), and water-cured samples (2 weeks). Figure 9 shows the total porosity results with respect to the different BFS replacement ratios after water curing for 2 weeks. It is revealed that the total porosity of paste samples tends to increase as the BFS replacement ratio increases because the proportion of cement content decreases and the reaction rate of BFS is delayed [22,23,24,25,26,27]. Moreover, in the case of the carbonated paste sample, the total porosity reduced with the level of carbonation, which shows the same tendency as the BFS cement paste, as seen in Figure 9. This is the same result as the cumulative pore volume. Figure 9 shows the total porosity after air curing. The capillary pore volume decreases as the BFS replacement ratio increases. This is due to the same reason as that of the water curing results. The pore structure of each compound and the total porosity results were determined by mercury intrusion porosimetry. It can be concluded that the pore volume could have been shifted towards a large pore volume by carbonation, and the total porosity of the carbonated samples was found to decrease.

## 4. Discussion of Pores Affecting Durability

**Figure 10:**
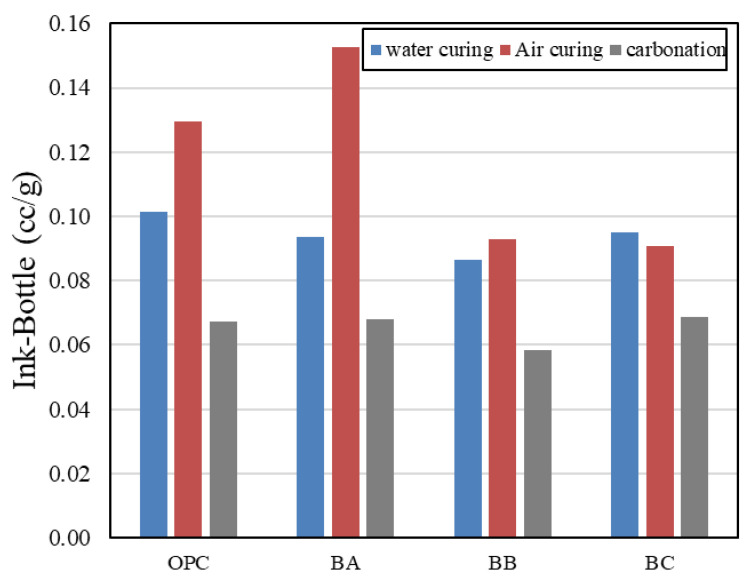
Ink-bottle volume of carbonated cement paste.

Figure 10 shows the ink-bottle pore volume in BFS-blended paste samples after 2 weeks of water curing. The method of measuring the total pore volume was carried out in the same way as in the previous paper [28,29]. According to the literature, the water in ink-bottle pores acts as a passage for carbon dioxide and influences carbonation. Furthermore, blocking ink-bottle pores lowers the carbonation rate [12]. Therefore, in this study, the higher BFS ratio increased the volume of the ink-bottle pores and tended to decrease the amount of lifting of the ink-bottle pores during the carbonation and air-curing processes. In addition, in the case of the air-cured test specimen, except for the BC specimen, the ink-bottle pore volume was higher than that of the water-cured specimen. In the carbonation test specimen, the volume of ink-bottle pores was significantly lower than that of the water-cured and air-cured specimens. In addition, as the replacement ratio of BFS increased, the volume of ink-bottle pores produced during air curing was lower, but there was no significant difference after carbonation. In the literature, the number of ink-bottle pores was determined to influence freezing and thawing resistance. In addition, several studies have shown that ink-bottle pores affect the increase in freeze–thaw resistance [30,31]. Therefore, it is determined that the number of ink-bottle pores reduced by carbonation affects the increase in freeze–thaw resistance.

**Figure 11:**
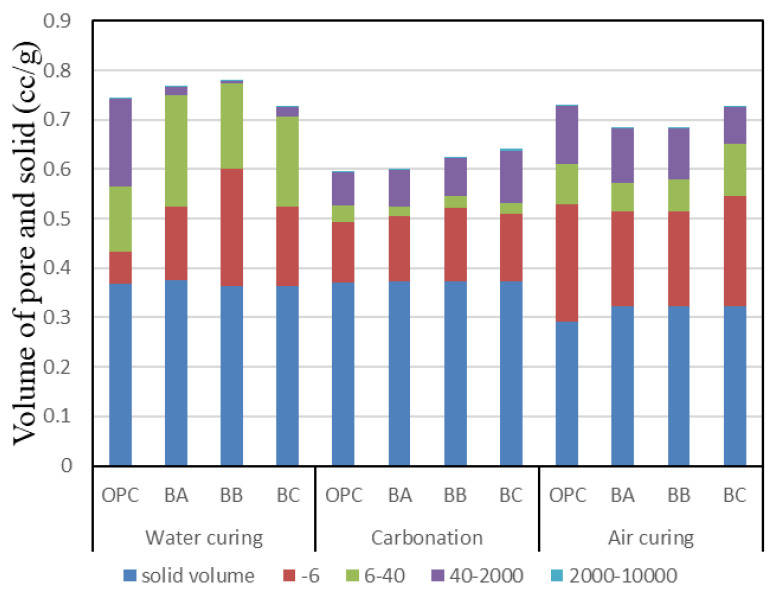
Pore structure of carbonated cement paste.

Figure 11 shows the pore structure of carbonated cement paste, including the solid volume, in BFS-blended paste samples after 2 weeks of water curing. According to Figure 11, the number of pores with sizes of 40–200 nm in the air-cured sample increases compared with that of the water-cured sample. In addition, when comparing the air-cured and the carbonation samples, it was confirmed that the number of pores with sizes of 40–2000 nm is reduced by carbonation, except for the BC specimen. As for the effect of BFS replacement, the higher the BFS replacement ratio, the higher the ratio of pores with sizes of 40–2000 nm due to carbonation. According to the literature, the number of pores in the size range of 40–2000 nm influences the freeze–thaw resistance. The freeze–thaw resistance increased as the number of pores in the size range of 40–2000 nm decreased [32,33]. The BC samples were an exception; as the amount of blast-furnace slag increased, the carbonated samples tended to show a lower number of pores with a diameter of 40–2000 nm than the air-cured samples. However, in this study, it was determined that there is little difference in the number of pores in the size range of 40–2000 nm in the mortar versus the concrete.

**Figure 12:**
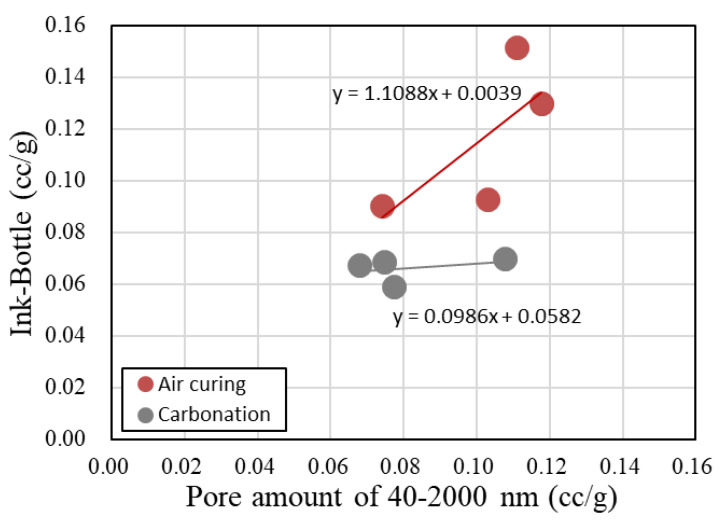
Relationship between 40–2000 nm pore volume and ink-bottle pore volume in carbonated cement paste.

Figure 12 shows the relationship between the 40–2000 nm pore volume and the ink-bottle pore volume in BFS-blended paste samples after 2 weeks of water curing. There is a significant disparity between the carbonated and air-cured samples. For the air-cured samples, as the total volume of the pores with diameters of 40–2000 nm increased, the total ink-bottle pore volume also tended to increase. However, the carbonated samples, except for the BC-2w sample, showed a lower ink-bottle pore volume and a lower volume of pores with diameters of 40–2000 nm than the air-cured samples. The freeze–thaw resistance was dependent on the pore structure, and the carbonated samples had a higher freeze–thaw resistance due to the effect of the low ink-bottle pore volume and the low volume of pores with diameters of 40–2000 nm.

**Figure 13:**
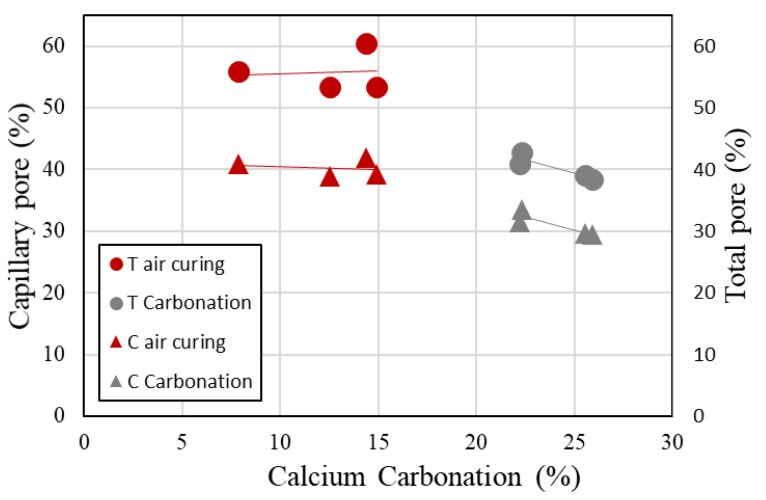
Relationship between the amount of calcium carbonation and the number of capillary pores and total pores in carbonated cement paste.

Figure 13 shows the result for the relationship between the amount of calcium carbonation with capillary pores and the total pore volume in BFS-blended paste samples after 2 weeks of water curing. At the same curing age, the total porosity and capillary porosity of the carbonation test sample decreases compared with that of the air-cured test sample. It can be said that the pore structures in the cement paste were reduced by carbonation.

These results show that the higher the replacement ratio of blast-furnace slag in the cement, the higher the amount of vaterite produced by carbonation. As described above, the lower the Ca/Si ratio, the greater the amount of vaterite produced. In this study, as the amount of Ca/Si decreased, the amount of vaterite increased as it replaced the blast-furnace slag. In the existing literature, it has been reported that when calcium hydroxide is converted to vaterite, a higher volume expansion ratio is displayed than when calcium hydroxide is converted to calcite. Therefore, it is assumed that the higher the replacement ratio of the blast-furnace slag, the smaller the pore size, which improves freeze–thaw resistance. In addition, when the concentration of hydroxides is high, the precipitation of Ca(OH)_2_ will be promoted and the replacement of Ca-rich sources accelerates both the initial and final setting time of the material [34,35].

## 5. Conclusions

In this study, the effect of carbonation on the properties of pore structure change and the chemical properties of BFS cement paste incorporating different BFS replacement ratios were investigated. The carbonation deterioration in cement paste was investigated in this research using mercury intrusion porosimetry, thermal analysis, and X-ray diffraction. However, this study is limited to the chemical composite and pore structure. The influence of carbonation on engineering properties, the dynamics and mechanisms of carbonation, and the differentiation of CH and CSH carbonation may be very important, so it is advisable to pursue further research. We will post these results in the future. The primary findings of this study can be summarized as follows:

1. The small pore distribution tends to increase as the BFS replacement ratio increases during water curing. Moreover, it was found that the carbonation of the cementitious composite could enhance the density of the cement paste samples. A shift towards a large pore distribution is caused after carbonation.
2. With respect to the influence of the DTG curve on each curing environment, it is notable that as the BFS replacement rate increased, the peak of the produced CC tended to decrease.
3. With respect to the influence of the amount of CH and C–S–H on carbonation, the thermal analysis of the carbonation amount from C–S–H increased with the BFS replacement ratios of the BB specimen and the BC specimen in the carbonation samples.
4. With respect to the influence of the cement mineral composite on the carbonated samples, it is notable that the amount of vaterite in the BFS-blended paste samples is larger than that of the N samples, and this phenomenon is primarily due to the low Ca/Si ratio of the C–S–H phase caused by BFS replacement.

In the future, we aim to clarify the reasons for the rapid pore structure change by incorporating BFS cement paste samples using the XRD/Rietveld quantification method to investigate the degree of mineral change after carbonation.

## Figures and Tables

**Table 1:** Chemical and physical properties of material.

	Chemical Composition (%)									Physical Properties	
	SiO_2_	Al_2_O_3_	Fe_2_O_3_	CaO	MgO	K_2_O	Na_2_O	SO_3_	LOI	Density (g/cm^3^)	Blaine Fineness (cm^2^/g)
OPC	21.06	5.51	2.69	65.47	1.66	0.4	0.24	1.91	2.28	3.17	3390
BFS	34.03	14.36	0.83	43.28	6.51	-	-	-	0.1	2.91	3930

**Table 2:** Mixture design of paste samples.

	W/C	Replacement Ratio (%)	
		OPC	BFS
N	0.65	100	-
BA		85	15
BB		55	45
BC		35	65

**Table 3:** Specific volume of paste samples.

	Water Curing	Air Curing	Carbonation
N	0.37	0.29	0.37
BA	0.37	0.32	0.37
BB	0.36	0.32	0.37
BC	0.36	0.32	0.37
